# Focus on 2010 ESC Congress

**Published:** 2010

## New ESC guidelines and data on dabigatran

In a major symposium on atrial fibrillation (AF), Prof John Camm, head of the Department of Cardiac and Vascular Sciences at St Georges, University of London, pointed out that atrial fibrillation cases are set to double as populations age. ‘One in four adults 40 years of age will develop atrial fibrillation in their lifetime. The consequences are tragic and devastating, with a five-fold increase in the risk of having a stroke’, he said. Stroke is also more likely to be severe and fatal in these patients; those who survive face persistent neurological deficits, persistent disability and poorer functional performance.^1^,^2^

The European Society of Cardiology (ESC) has now issued revised practice guidelines for the management of atrial fibrillation, including guidance on the role of a novel oral treatment, dabigatran etexilate, for the prevention of stroke and systemic embolism in patients with atrial fibrillation.

Dr Jeffrey Friedman, therapeutic head of cardiovascular products at Boehringer Ingelheim in the USA confirmed that the US Food and Drug Administration (FDA) has granted a priority review designation for dabigatran etexilate for the prevention of stroke in AF. A priority review designation is given to new drugs that are expected to offer major advances in treatment, or provide a treatment where no adequate therapy exists. (An FDA advisory committee met on September 20 to review and discuss dabigatran etexilate data.)*

In addition to the USA, the registration process for dabigatran etexilate is underway in Europe, Japan and other countries. The company expects to receive marketing authorisation for dabigatran etexilate in these countries by the end of 2010 or the beginning of 2011.

Dr Friedman pointed out that Boehringer Ingelheim has a long-term commitment to stroke prevention, with the development of Actilyse in 1982, Asasantin retard in the 1990s, and more recently, Micardis for the prevention of stroke and myocardial infarctions in the early 2000s. ‘Telmisartin is still the only ARB with a broad claim for cardiovascular protection, based on the ONTARGET studies’, Dr Friedman noted.

Discussing the latest RELY results,3 Dr Jonas Oldgren from the Uppsala Clinical Research Centre, pointed out that all centres, even those with poorer INR control determined from their patients’ time-in-treatment data, achieved better results using dabigatran than warfarin in the reduction of stroke and systemic embolism.

As might be expected in the poorer warfarin-control setting, both dosages of dabigatran demonstrated superior advantages over warfarin in the reduction of secondary outcomes, such as the composite of all cardiovascular events, total mortality and major bleeding. The secondary outcome results in centres with better INR control were comparable between dabigatran and warfarin. ‘Local standards of care do therefore affect the benefits of switching to new treatment strategies’, Prof Oldgren said.

For each patient involved in the warfarin arm of the RELY trial, the quality of warfarin treatment was calculated by establishing the time in therapeutic range (TTR). The average individual time in therapeutic range (iTTR; with a target INR of 2.0–3.0) for patients randomised to warfarin was 64%, which is a similar level of control to that seen in recently published prospective, randomised trials.

There were considerable variations in TTR among the trial centres across the 44 participating countries. In the present sub-group analysis, each centre’s average TTR (cTTR) was calculated as the average of all individual patients’ TTRs in the warfarin group.

The distribution of cTTRs across study centres was investigated and interquartile limits were identified. The quartiles of cTTR for the warfarin patients were < 57.1%, 57.1–65.5% and > 72.62%. Outcomes were assessed across the three treatment arms (dabigatran etexilate 110 mg BID, 150 mg BID, and warfarin).

Results demonstrated that:^3^xref>

• Dabigatran etexilate 150 mg BID was superior to warfarin in the reduction of stroke and systemic embolism, independent of the level of centre-based INR control.
• Dabigatran etexilate 110 mg BID was similar to warfarin in the reduction of stroke and systemic embolism, with lower rates of major bleeding, independent of the level of centre-based INR control.
• Both dosages of dabigatran etexilate were superior to warfarin in terms of intracranial haemorrhage (ICH), irrespective of centre-based INR control.

For further information on dabigatran please contact Sue Thomas (Medical Information, Boehringer Ingelheim) on (011) 348-2514.

## *Stop press

FDA meeting on dabigatran

One of cardiology’s fondest wishes moved closer to fulfillment as an FDA advisory panel unanimously recommended approval of a potential replacement for warfarin in one of the most common heart disorders. Barring any unforeseen damning revelations about the drug, the FDA’s approval of the oral thrombin inhibitor dabigatran (Pradaxa, Boehringer Ingelheim) for stroke prevention in atrial fibrillation is all but certain.

The panel, with its nine voting members, made the decision based on what’s generally seen as the well-designed, solidly executed, 18 000-patient Randomized Evaluation of Long-Term Anticoagulant Therapy (RELY) trial, which showed dabigatran was non-inferior to warfarin at a lower dosage and superior at a higher one for preventing thromboembolic stroke in paroxysmal or permanent AF.

Debate among the advisory panel throughout the day was tame; there were few criticisms of the RELY trial’s design, little disappointment in the results, and clear enthusiasm for replacing warfarin in such a widespread indication.

The panel wasn’t charged with voting on a question that provided some of the only suspense throughout the day; whether the approval should include only the higher dose, which demonstrated superiority in RELY, or perhaps both dosage levels. They voted informally nonetheless, with those expressing a preference for both dosages (generally wanting to give clinicians more flexibility) edging out those favouring only the higher dose. Some of the latter panelists felt the lower dose would become the default for clinicians concerned about safety but at the expense of efficacy.

Warfarin is distinguished as being one of the oldest, most widely used, most effective, and most disliked drugs in cardiology: it dramatically cuts ischaemic stroke risk in AF but generally requires tight anticoagulation monitoring to get the dosage right, which can be arduous for patients and the healthcare system. Patients must also banish a lot of healthy, vitamin K-containing foods from their diet.

The kicker with dabigatran, even when ‘non-inferior’ to warfarin, is that it doesn’t require anticoagulation monitoring, or major diet changes, for that matter. And, as the trial suggested and some panelists agreed, at the higher dose it seems more stroke-preventive than warfarin.

As previously reported by heartwire, RELY compared the two dabigatran dosage levels against a conventional warfarin regimen for the strokeprevention indication. Dabigatran was non-inferior to warfarin at the lower dosage and superior at the higher one, the latter achieving a 34% decline in risk (p < 0.001) over a median of two years.

Source: theheart.org
